# *Dactylopius opuntiae* [Cockerell] Could Be a Source of Antioxidants for the Preservation of Beef Patties

**DOI:** 10.3390/insects14100811

**Published:** 2023-10-13

**Authors:** Othoniel H. Aragon-Martinez, Flavio Martinez-Morales, Marco M. González-Chávez, Santiago de J. Méndez-Gallegos, Rodolfo González-Chávez, Juan C. Posadas-Hurtado, Mario A. Isiordia-Espinoza

**Affiliations:** 1 Laboratorio de Productos Naturales, Facultad de Ciencias Químicas, Universidad Autónoma de San Luis Potosí, San Luis Potosí 78210, Mexico; 2 Departamento de Farmacología, Facultad de Medicina, Universidad Autónoma de San Luis Potosí, San Luis Potosí 78210, Mexico; martinej@uaslp.mx; 3 Colegio de Postgraduados, Campus San Luis Potosí, Posgrado en Innovación en Manejo de Recursos Naturales, Salinas de Hidalgo 78622, Mexico; jmendez@colpos.mx; 4 Facultad de Ciencias Químicas, Universidad Autónoma de San Luis Potosí, San Luis Potosí 78210, Mexico; rodolfo.gonzalez@uaslp.mx (R.G.-C.); j_c247@hotmail.com (J.C.P.-H.); 5 Departamento de Clínicas, División de Ciencias Biomédicas, Instituto de Investigación en Ciencias Médicas, Centro Universitario de los Altos, Universidad de Guadalajara, Tepatitlán de Morelos, Jalisco 47620, Mexico; mario.isiordia162@yahoo.com

**Keywords:** wild cochineal, insect pest, meat preservative, lipid oxidation, antioxidant capacity

## Abstract

**Simple Summary:**

*Dactylopius opuntiae*, known as wild cochineal, is an insect pest of cactus crops in several countries, which produces important economic losses in this agricultural sector. The objective of our study was to use an agricultural pest as a preservative source for the beef meat industry, which is in search of a natural preservative to replace the approved synthetic additives, such as butylated hydroxytoluene. Our study showed the usefulness of a *D. opuntiae* extract, which was obtained using an accessible procedure, to preserve the color and reduce the oxidation process on beef patties stored under refrigeration. The beneficial actions on meat parameters were caused by the presence of carminic acid, which is a metabolite found in this insect with antioxidant properties, where that ability was supported by using two synthetic free radicals scavenging assays. Our study provides a feasible, solid–liquid extraction to obtain an antioxidant preservative with direct application on beef patties and supports their use by the improvement of meat acceptability criteria. The importance of our study lies in the fact that beef meat has a crucial role in human nutrition, and ground beef constitutes 64% of the meat consumed by humans.

**Abstract:**

*Dactylopius opuntiae* is an insect pest that contains at least carminic acid, which has antioxidant properties. Since there is a relationship between the antioxidant ability and preservative action of compounds applied to meat products, the purpose of this study was to evaluate the antioxidant activity and usefulness of a *D. opuntiae* extract for beef patty preservation. The insects were bred and processed to obtain a liquid extract. For the extract, its carminic acid content, antioxidant activity against two free radicals, and actions on food quality parameters were determined. The *D. opuntiae* dry powder contained 2.91% *w/w* carminic acid, while the liquid extract exhibited an IC50 value of 3437.8 ± 67.8 and 19633.0 ± 674.5 µg/mL against the DPPH and ABTS radicals. Nevertheless, these antioxidant actions were lower than those found in a *D. coccus* extract. The *D. opuntiae* extract improved in a short time the redness and yellowness, eliminated the unfavorable effect of their vehicle on the MetMb level, and greatly reduced the TBARS formation. For the first time, an extract of *D. opuntiae* was applied to beef patties, and its beneficial antioxidant action on meat acceptance parameters was confirmed, which has potential commercial applications.

## 1. Introduction

*Dactylopius* (Hemiptera: Coccoidea: Dactylopiidae) is an insect that feeds on the sap of prickly pears of the Opuntia species. *Dactylopius* is the main source of carminic acid, which is a red dye used in cosmetics, drugs, foods, and textile products. The genus *Dactylopius* comprises 11 species, where only *Dactylopius coccus* is used for the industry as a source of carminic acid, whereas *Dactylopius opuntiae*, known as wild cochineal, is considered an invasive pest of the Opuntia species [1,2]. The nymph and female adult stages of *D. opuntiae* produce chlorosis and premature dropping of cladodes and fruits, which in some cases lead to plant death. The damage caused by this pest is severe and generates important economic losses in cactus crops [2]. *D. opuntiae* insects have an extensive geographical distribution in 28 countries, such as Australia, Brazil, India, France, Mexico, South Africa, and the United States, among others. Meanwhile, *D. coccus* insects are found in 19 countries, such as Argentina, Chile, Ecuador, Egypt, India, Mexico, and South Africa, among others [3].

For carminic acid, some biological actions have been described, such as the suppression of tumors, antioxidant activity, and attenuation of the progression of fatty liver disease [4]. Meanwhile, carmine, a chelate of carminic acid with various metal ions extracted from *D. coccus*, is used as a food additive in the meat industry to improve color stability. However, some factors limit their use, such as aluminum exposure and reports of allergic reactions in consumers, and substitutes for carmine are currently being sought [5]. Similarly, alternatives for the use of butylated hydroxytoluene (BHT), which is a common meat preservative approved for human use in food by the United States regulations, are desirable since some deleterious effects are reported for this phenolic compound in animal models under a prolonged and certain level of consumption, including toxic nephrosis and the development of liver tumors [6].

Ground beef constitutes 64% of all the meat consumed by humans, and beef meat has a crucial role in the human diet as a source of proteins, minerals, vitamins, and other nutrients to maintain a healthy state in humans [7,8]. Commonly, a period of refrigeration of ten days is required to distribute meat products to retail outlets [6]. Since two crucial oxidative processes, lipid and protein oxidation, occur during the period of refrigeration of the meat, compounds with antioxidant properties are useful to preserve these products, such as beef patties. These compounds can be added in a single form or in a mixture to beef patties, where the replacement of synthetic preservatives such as BHT by compounds obtained from natural sources is desirable [6]. The antioxidant abilities of compounds can be tested with various assays in vitro that expose the compounds to different free synthetic radicals, such as the 2,2′-diphenyl-1-picrylhydrazyl (DPPH) or 2,2′-azino-bis 3-ethylbenzothiazoline-6-sulfonic acid (ABTS) radical. The scavenging activity of the antioxidant against the radical is monitored through the inherent spectral properties of the intact radical [9,10].

To our knowledge, an extract rich in carminic acid and obtained from an insect pest, such as *D. opuntiae*, has never been applied to meat products. In fact, there are efforts to control or eliminate this pest, but their usefulness has not been explored. Therefore, the present study aimed to evaluate the carminic acid content, antioxidant properties, and usefulness of a *D. opuntiae* extract for the preservation of beef patties.

## 2. Materials and Methods

### 2.1. Reagents

Allopurinol, ascorbic acid, carminic acid (90% of purity), BHT, carmine (contains 42% of carminic acid), sodium carbonate, sodium hydroxide, acetic acid, citric acid, DPPH, potassium persulfate, ABTS diammonium salt, thiobarbituric acid, 1,1,3,3-tetraethoxypropane, hydrochloric acid, and trichloroacetic acid were obtained from Sigma-Aldrich (St. Louis, MO, USA).

### 2.2. Insect Breeding

Cladodes of *Opuntia ficus-indica* cv. Rojo pelón from six to eight months and *D. opuntiae* [Cockerell] were obtained from an orchard belonging to the Colegio de Postgraduados, Campus San Luis, Salinas de Hidalgo, S.L.P., Mexico. Meanwhile, *D. coccus* was acquired from the Esquivel Hermanos company, Santa Clara, Jerez, Zacatecas, Mexico. In a previous study, the identification of these *Dactylopius* specimens was performed using microscopic features [11], and since that date, the specimens have been reproducing. The cladodes were cleaned to remove dust and spines, and then they were placed inside a greenhouse to allow for healing. Subsequently, the cladodes were placed in a recipient containing wet soil and exposed to *D. coccus* or *D. opuntiae* nymphs for 48 h. Under controlled humidity (40%) and temperature (23.5 °C) conditions, the infested cladodes were placed in a box covered with a fine mesh to produce adult female insects [12].

### 2.3. Carminic Acid Extraction

*D. opuntiae* adult female insects alongside their wax were collected from the infested cladodes, and then, these samples were dried at 40 °C, powered with a blender, and stored in polypropylene recipients at room temperature while avoiding sunlight. To obtain the cochineal extract, a conventional solid–liquid extraction was performed with some modifications [13]. A 433 mg/mL citric acid solution was prepared using distilled water, and then, the powdered samples (M6 = 0.21 g, M5 = 0.42 g, M4 = 0.84 g, M3 = 1.69 g, M2 = 3.34 g, or M0 = 0.00 g) were mixed with 10 mL of the citric acid solution. The mixture was incubated at 50 °C for 30 min, cooled at 4 °C for 14 min, and centrifuged at 1500 rpm for 10 min at room temperature. After centrifugation, three phases were identified, and the intermediate layer was collected. The superior and inferior layers were cochineal and wax remains. Then, 1.14 g of sodium carbonate was gradually added to the layer collected. This mixture was maintained at room temperature until the formed foam disappeared completely, and then, the mixture was centrifuged again (1500 rpm for 10 min at room temperature). Two layers were observed after this second centrifugation, and the lower layer was collected, weighed, and monitored their pH value by using an Accumet^®^ AR15 device (Fisher Scientific, Pittsburgh, PA, USA) and stored at −20 °C. To compare the extracts obtained, the antioxidant capacity against the DPPH radical and recovery of the liquid extract were considered. The DPPH radical scavenging assay is described in detail below. For the samples containing carminic acid (*D. coccus* adult female insects, carmine, and carminic acid), this same procedure was performed, but the mass of the carminic acid reagent used was 2.67% of the mass used for the M6-M2 samples. BHT was not processed in this extraction since it is used directly on meat products [14].

### 2.4. Carminic Acid Quantification in Cochineal Extracts

This analysis was performed as described previously [15] but with modifications. First, stock solutions of 236 µg/mL carminic acid and 250 µg/mL allopurinol were prepared in 0.2 N acetic acid and 15 mM sodium hydroxide, respectively. Prior to the analyses, the extract samples (0.00 or 1.69 g of cochineal powder processed as described above) were diluted 1:100 *v/v* with distilled water and fortified with allopurinol as the internal standard (81.3 µg/mL). Subsequently, calibrators containing carminic acid (0.59–118 µg/mL) and allopurinol (81.3 µg/mL) were prepared in distilled water. Thus, 200 µL of the diluted extract or calibrator samples were mixed with 200 µL of methanol and then centrifuged at 12,100 rpm for 10 min at 4 °C. The supernatant (120 µL) was placed in a vial insert inside an amber glass tube for chromatographic analysis. A Waters 600 liquid chromatography system consisting of a quaternary pump with degasser, autosampler, thermostated column compartment, and diode array detector was used for analyses (Waters Corporation, Milford, MA, USA). For the chromatographic separation, a 120 mm long YMC-PACK Pro C18 column was used with a 5 µm particle size and a 4.6 mm internal diameter (Agilent Technologies, Santa Clara, CA, USA). The column compartment was maintained at 40 °C during the separation, and an isocratic mobile phase was used, which was an acetic acid solution (3.49 g/L) plus methanol at a ratio of 60:40 *v/v*. The analysis was performed with an injection volume of 10 µL of the supernatant sample, a rate of 1 mL/min, monitoring at 274 and 254 nm for carminic acid and allopurinol, 8 min of run time, and drawing the spectra from 190 to 400 nm. For each chromatographic peak identified by retention time and spectrum record, the carminic acid concentration in the sample was obtained by interpolating their response into the curve performed with the calibrators. The response was the ratio of the peak areas monitored at 274/254 nm, and the concentration was multiplied by the dilution factor. Finally, the percentage of carminic acid in the powdered sample was calculated using the results expressed as µg of carminic acid/mL, the volume of solution, and the mass of the cochineal powder used.

### 2.5. Antioxidant Activity of Samples against the DPPH Radical

This assay was performed as described previously [10] but with some modifications. Fifty microliters of each sample were mixed with 200 µL of 450 µmol/L DPPH solution, and then, the mixture was incubated at 30 °C for 20 min. Subsequently, each mixture was centrifuged at 1500 rpm for 5 min at 15 °C. Then, 150 µL of each supernatant was placed per well in a 96-well plate (Costar^®^ 3595, Corning Incorporated, New York, NY, USA). The absorbance response was monitored at 517 nm for each sample in wells using the Cytation™ 3 microplate reader and Gen5™ software version 2.06 (Biotek Instruments Inc., Winooski, VT, USA). The results of this assay were expressed as the percentage inhibition of the radical or µg of ascorbic acid equivalents (AAE)/mL of sample. The percentage of inhibition against the DPPH radical was calculated using the absorbance values of the blank (distilled water) and experimental samples. The value expressed in µg AAE/mL of samples was obtained by interpolating the response of diluted samples into ascorbic acid calibration curves ranging from 6.0 to 120.0 µg/mL. For these experiments, ascorbic acid and distilled water were included as the positive and negative controls.

### 2.6. Antioxidant Activity of Samples against the ABTS Radical

This assay was carried out as described by Gonzalez-Rivera and collaborators [9] but with some modifications. First, the radical was produced with the reaction between a 7.0 mmol/L ABTS diammonium salt solution and 2.4 mmol/L potassium persulfate solution during 16 h in darkness. Subsequently, this mixture (1.14 mL) was diluted with deionized water (48.86 mL) to obtain a 95.7 µmol/L ABTS radical solution. Thus, 3 µL of each sample was mixed with 300 µL of the ABTS radical solution and then incubated at 30 °C for 4 min. Subsequently, each mixture was centrifuged at 1500 rpm for 5 min at 15 °C. Each supernatant was placed in a well of a 96-well plate to monitor their absorbance response at 730 nm using the same microplate reader and software mentioned above. The percentage of inhibition against the ABTS radical was performed as was described for the DPPH radical. Again, ascorbic acid and distilled water were included as positive and negative controls of the assay.

### 2.7. Preparation of Beef Patties

The meat preparation was carried out as described previously [6]. Fresh beef pulp was purchased from a local supermarket, transported to the laboratory, and ground in a meat grinder fitted with a 4.5 mm grind plate (Tartare, Metaltex International, Molsheim Cedex, France). Four formulations were prepared: meat without additive (Co); meat with 1.216 mL/Kg M0 extract added (M0); meat with 100 mg/Kg BHT added (BHT); and meat with 1.216 mL/Kg M3 extract added (M3).

Each preparation was mixed at low speed for 2 min in a mixer (Model 64650, Hamilton Beach Brands Inc., Glen Allen, VA, USA), and then a 100 g portion of each meat sample was made into an 11.5 cm diameter and 1.5 cm in height burger. Beef patties were placed in polyethylene bags and stored at 4 °C in the absence of light. Meat samples were analyzed for color, antioxidant activity against the DPPH radical, metmyoglobin (MetMb) content, and thiobarbituric acid reactive substance (TBARS) value on days 0, 6, and 12. The evaluation at day 0 was performed within a time period of 30 min with a temperature of 4 °C after the meat preparation.

The BHT concentration used was the concentration recommended for use with meat products [13]. The concentration and volume of the cochineal extract were determined on the basis of their antioxidant IC50 value against the DPPH radical, where the used extract presented the same antioxidant activity found in the selected BHT concentration [6]. The volume used of the M0 and M3 extracts was inferior to the volume of oily preservatives used in ground beef [14].

### 2.8. Color Measurement

This color evaluation was performed as described by Gallegos and collaborators [15]. A Color Muse colorimeter (Variable Inc., Chattanooga, TN, USA) was directly placed on the surface of the beef patty in areas without fat or connective tissue at four different points. The CIE Lab system, which determines the L*, a*, and b* values, was applied for each point, where these values were assessed as a measure of lightness, redness, and yellowness, respectively. For this color system, the term redness represents the color scale from +60 for red to −60 for green. Meanwhile, the term yellowness represents the color scale from +60 for yellow to −60 for blue [16]. Before any measurement, the colorimeter was calibrated with a standard white plate provided by the same manufacturer. For each sample, a mean value for each CIE Lab parameter was calculated.

### 2.9. Antioxidant Activity of Beef Patties

This procedure was performed as described previously [6] but with some modifications. A beef patty sample (0.50 g) was placed in a tube containing 500 µL of deionized water, mixed for 1 min, and centrifuged at 4000 rpm for 10 min at 4 °C. Subsequently, 50 µL of the supernatant or ascorbic acid calibrators were processed using the DPPH radical scavenging assay as described above. Considering data on the volume and mass of the meat sample used, the results are expressed as µg AAE/g of the sample.

### 2.10. Metmyoglobin Content

The measurement of MetMb was conducted according to Mtibaa and collaborators [17]. A sample of beef patty (0.2 g) was placed in a tube containing 1 mL of 40 mM cold phosphate buffer solution at pH 6.8, mixed for 1 min, and then, the mixture was kept at 4 °C for 1 h and centrifuged at 6800 rpm for 30 min at 4 °C. The supernatant was filtered through a 0.45 µm pore size filter unit (Millipore Corporation, Bedford, MA, USA). Two hundred microliters of the filtrate were placed into the wells of a 96-well plate to record the absorbance response at 572, 565, 545, and 525 nm using the same microplate reader and software mentioned for the antioxidant assays. Using the four absorbance values, the MetMb percentage was calculated as described previously [6].

### 2.11. Thiobarbituric Acid Reactive Substance Value

The determination of TBARS was performed as described by Martinez-Morales and collaborators [6]. A sample of the beef patty (0.17 to 0.19 g), 1 mL of 50 g/L trichloroacetic acid, and 6 µL of 1g/L BHT were mixed for 1 min. This mixture was centrifuged at 4400 rpm for 10 min at 18 °C. A volume of the supernatant or malondialdehyde calibrator (250 µL) was mixed with 150 µL of 0.8% thiobarbituric acid, and then, this mixture was incubated at 75 °C for 30 min and kept at 4 °C for 3 min. Two hundred microliters of the mixture were placed in one well of a 96-well plate to read its absorbance at 532 nm using the same microplate reader and software mentioned for the antioxidant assays. The TBARS value was calculated using the calibration curve of malondialdehyde, where malondialdehyde calibrators (range from 0.019 to 2.400 µg/mL) were prepared from a 239 µg/mL malondialdehyde stock solution. This stock solution was obtained from the degradation of 1,1,3,3-tetraethoxypropane, which was induced by their exposition to 0.1 N hydrochloric acid in a boiling water bath. Considering the volume and mass of samples used, the results were expressed as µg TBARS/g of meat.

### 2.12. Data Analysis

Data are presented as the mean and standard deviation. The analyses of these obtained data from the selection of mass for extraction (*n* = 3) and antioxidant activity assays (*n* = 5) were performed using a one-way ANOVA and Tukey post-test. Meanwhile, data obtained from the meat studies (*n* = 6) were analyzed using a two-way ANOVA with Bonferroni post-test. A *p*-value of <0.05 was considered statistically significant. The calculation of IC50 values and statistical analyses were performed using the GraphPad Prism 5 software (San Diego, CA, USA).

## 3. Results

### 3.1. Extraction Outcomes

**Figure 1:**
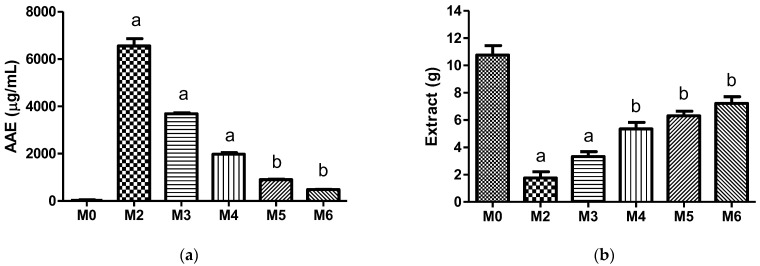
Results of the tests applied to the *D. opuntiae* samples obtained from the different procedures (*n* = 3): (**a**) Antioxidant capacity against the DPPH radical; (**b**) Recovered mass of the liquid extract. Lower case letters (a and b) indicate differences as follows: ^a^ *p* < 0.0001 versus all other procedures and ^b^ *p* < 0.0001 versus M2, M3 and M4 procedures, and *p* < 0.05 versus M0, M5 and M6 procedures.

The antioxidant capacity of the *D. opuntiae* liquid extract was increased as the mass of the cochineal powder used was increased (Figure 1a). However, the recovery of the liquid extract was more difficult and was diminished as the mass of powder was increased (Figure 1b). Similar results were obtained from the extraction of *D. coccus*, carmine, and carminic acid. For all the evaluated samples (*D. opuntiae*, *D. coccus*, carmine, and carminic acid extracts), the pH of the liquid extracts was 4.54 ± 0.36 (*n* = 72).

### 3.2. Carminic acid Content

*D. opuntiae* and *D. coccus* dry powders exhibited 2.91 ± 0.36 and 4.03 ± 0.32% *w/w* of carminic acid (*n* = 6 for each powder), while carminic acid was not detected in the M0 extracts (*n* = 6). During the chromatographic analysis, the retention time and wavelength absorption maxima value for carminic acid and allopurinol were 3.1 and 1.7 min at 275 and 250 nm, respectively.

### 3.3. Antioxidant Activity of Samples against the Synthetic Radicals

**Figure 2:**
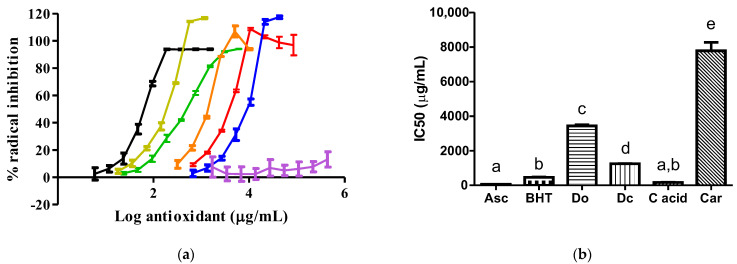
Actions of samples against the DPPH radical: (**a**) Graphics of the radical inhibition (for each curve, six to nine points were plotted with *n* = 5 for each point); (**b**) IC50 values of each sample (*n* = 5). A different letter means that there is statistical difference (*p* < 0.0001). The black, olive, green, orange, red, blue, and violet line represents ascorbic acid (Asc), carminic acid (C acid), BHT, *D. coccus* (Dc), *D. opuntiae* (Do), carmine (Car) and the M0 extract, respectively.

As shown in Figure 2, a higher antioxidant potency against the DPPH radical was exhibited by ascorbic acid and pure carminic acid, compared with the rest of the tested samples. *D. opuntiae* had a superior antioxidant potency to carmine and the M0 extract.

**Figure 3:**
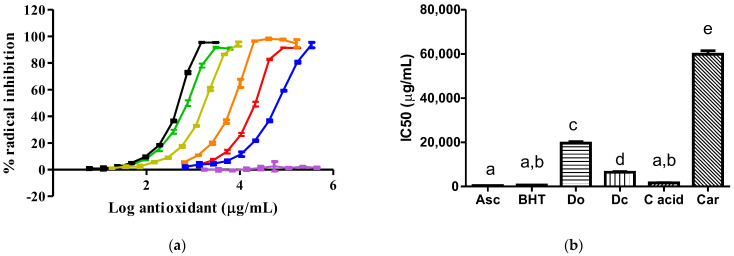
Actions of samples against the ABTS radical: (**a**) Plots of the radical inhibition (for each curve, eight to ten points were plotted with *n* = 5 for each point); (**b**) IC50 values of each sample (*n* = 5). A different letter means that there is statistical difference (*p* < 0.0001). The black, olive, green, orange, red, blue, and violet line represents ascorbic acid (Asc), carminic acid (C acid), BHT, *D. coccus* (Dc), *D. opuntiae* (Do), carmine (Car) and the M0 extract, respectively.

For the ABTS radical inhibition, a higher antioxidant potency was exhibited by ascorbic acid, pure carminic acid, and BHT, compared with the rest of the tested samples (Figure 3). Again, the *D. opuntiae* extract had a superior antioxidant potency to carmine and the M0 extract.

### 3.4. Effects of Supplementation on Beef Patties

Table 1 shows data obtained from the meat color evaluation (*n* = 6). *D. opuntiae* extract did not modify the lightness and produced an instantaneous beneficial action on redness and yellowness, but these two beneficial actions were lost on day 6. On the other hand, the BHT produced a detrimental action on lightness and a more pronounced increase in the redness and yellowness in beef patties compared with the *D. opuntiae* extract. The M0 additive had a negative effect on lightness and a rapid benefit on redness and yellowness, but this was not maintained over time. During the analyses, the sources of variation were the type of additive (*p*< 0.0001 for lightness and redness, and *p* = 0.0174 for yellowness), time (*p* < 0.0001 for redness and yellowness), and the interaction between these two factors (*p* = 0.0025 or <0.0001 for redness or yellowness).

**Figure 4:**
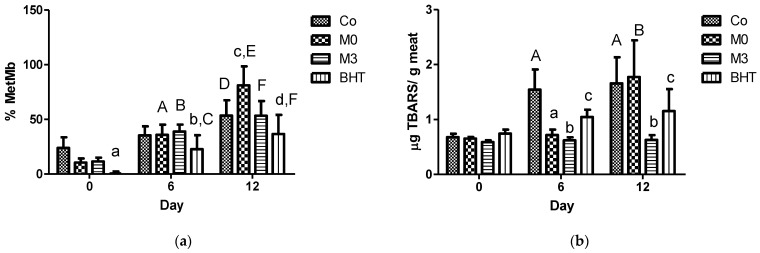
Effects of additives on the beef meat (*n* = 6): (**a**) MetMb content; (**b**) TBARS content. Lowercase letters indicate differences within the same day, while uppercase letters indicate differences between days. For graphic (**a**): ^a^ *p*-value < 0.05 versus Co; ^b^ *p*-value < 0.05 versus M3; ^c^ *p*-value < 0.001 versus all groups; ^d^ *p*-value < 0.05 versus Co and M3; ^A^ *p*-value < 0.001 versus their same group at day 0 and 12; ^B^ *p*-value < 0.001 versus their same group at day 0; ^C^ *p*-value < 0.01 versus their same group at day 0; ^D^ *p*-value < 0.01 versus their same group at day 0 and 6; ^E^ *p*-value < 0.001 versus their same group at day 0; and ^F^ *p*-value < 0.001 versus their same group at day 0. For graphic (**b**): ^a^ *p*-value < 0.001 versus Co; ^b^ *p*-value < 0.001 versus all groups; ^c^ *p*-value < 0.01 versus Co and M0; ^A^ *p*-value < 0.001 versus their same group at day 0; and ^B^ *p*-value < 0.001 versus their same group at day 0 and 6.

The antioxidant meat capacity did not show significant changes between the groups and times evaluated (Table 1, *n* = 6). Except on day 12, the BHT group exhibited a superior action against the DPPH radical than the Co group, but the overall analysis did not identify the sources of variation (*p*-values > 0.18). BHT and M0 had a beneficial and detrimental action on MetMb content, respectively, where the components of the *D. opuntiae* extract in M3 removed the unfavorable effect of M0 in meat, as is shown in Figure 4a. The additive used (*p* < 0.0001), time (*p* < 0.0001), and interaction between them (*p* = 0.0001) were identified as sources of variation for the MetMb percentage in meat.

The *D. opuntiae* extract produced an important reduction in TBARS formation in meat compared with the other additives (Figure 4b). In addition, BHT and M0 had a beneficial action on TBARS, but in a minor way compared with the extract. For the analysis of TBARS, the additive used, time, and interaction between these factors were the sources of variation (*p*-values < 0.0001).

## 4. Discussion

The M3 procedure was selected as the method for carminic acid extraction from dried *D. opuntiae* powder. This procedure produced an acceptable balance between the antioxidant ability and recovery of the extract (Figure 1). Our M3 extraction was an easy and accessible procedure because it only involved a conventional solid–liquid extraction [13], and the liquid extract was used directly in meat products. This type of methodology is desirable for the agricultural sector growing in communities with economic restrictions, such as Mexico [18]. Other types of procedures can be applied to extract carminic acid from insect powders, but those involve high costs and sophisticated equipment, such as the apparatus for supercritical fluid extractions [13]. To our knowledge, this is the first time that the carminic acid content of an insect pest product, such as *D. opuntiae* powder, has been reported. Our carminic acid content is inferior to the range of 3.9 to 25.7% reported for powder and liquid food additives derived from *D. coccus* [19]. The difference in carminic acid content may be caused by the application of a one-step extraction method or different light exposure times of insects. It is well known that using additional extractions on a sample increases the recovery of the active compound. However, additional steps consume more time and resources. Furthermore, light exposure is a factor that influences red dye production in *D. coccus* [20].

Since the antioxidant activity of compounds has a strong relation with their preservative properties observed in meat products [6], we evaluated the antioxidant capacities of our extract against two synthetic free radicals, such as the DPPH and ABTS radicals (Figure 2 and Figure 3). For the first time, we reported that a *D. opuntiae* extract exhibited antioxidant activities against the two synthetic radicals, and these activities were superior to those observed in the carmine and M0 samples. The antioxidant properties found in our *D. opuntiae* and carmine extracts are mainly due to the presence of carminic acid in these samples. Carminic acid is known to be a substance with free radical scavenging properties [4]. Then, the *D. opuntiae* extract can be used instead of carmine, where the disuse of carmine in foods is a present health trend [13]. It is important to mention that the antioxidant abilities of the *D. opuntiae* extract had a lower potency than those produced by ascorbic acid, pure carminic acid, BHT, and *D. coccus* extract. The minor antioxidant capacity of the *D. opuntiae* extract compared with that of the *D. coccus* extract was caused by the lower content of carminic acid in the *D. opuntiae* powder than that found in the *D. coccus* powder, as mentioned in Section 3.2. Coinciding with our data, ascorbic acid is recognized as a substance with a higher antioxidant capacity than BHT [21], carminic acid is a recognized antioxidant as mentioned above, and *D. coccus* extract has a potential antioxidant activity caused by its carminic acid content in insects [20].

Using our information on the antioxidant IC50 values of the extract and BHT against the DPPH radical, the usefulness of the *D. opuntiae* extract as a meat preservative was evaluated, and their activity was compared with that of BHT (Table 1). The BHT compound is an approved and common preservative used in meat and meat products [14]. The evaluation of color parameters, such as lightness, redness, and yellowness, is important since this is a predictor of the acceptability of a meat product by consumers. All these parameters decrease as refrigeration time increases, and the redness has a key role as an acceptability predictor [6]. In this manner, our data show that the *D. opuntiae* extract produced a superior benefit on the lightness compared with BHT alongside an instantaneous improvement in the redness and yellowness compared with meat free of preservatives. Our information cannot be compared with previous data because there are no earlier studies of a *D. opuntiae* extract applied to meat products. However, data on carmine applied in mortadella and sausage show that their use produces a reduction in the lightness and yellowness with an increase in the redness in comparison with the additive-free group during the storage period [5,22].

The antioxidant status of the beef patties did not show an improvement with the addition of the *D. opuntiae* extract. Again, there is no information related to this outcome in the literature. A possible explanation for this situation is that the antioxidants derived from the extract were consumed by the free radicals generated in the meat matrix during their storage, and then, their presence was not able to increase the overall antioxidant capacity measured by the DPPH scavenging assay [23]. On the other hand, the improved antioxidant capacity in meat samples when using BHT agreed with previous data reported for this compound added to the same food product [6]. The discoloration of meat is produced by myoglobin oxidation and MetMb formation, where this latest variable is related to the consumer rejection of meat products when a level of more than 40% is reached [17]. Commonly, the MetMb level in meat products increases as the storage time is prolonged [6]. In this case, only meat fortified with BHT maintained MetMb levels below 40% throughout the storage period. On the other hand, the *D. opuntiae* extract, M0, and additive-free meat maintained an acceptable MetMb level at days 0 and 6 (Figure 4a). At day 12, the carminic acid present in the *D. opuntiae* extract reduced the detrimental effect of the M0 on the MetMb level of meat but outside of the criteria for consumer acceptance. This is the first report on the effects of a *D. opuntiae* extract on the MetMb content in beef patties. Meanwhile, the undesirable effect of M0, which contains sodium citrate that was produced by the reaction between the citric acid and sodium carbonate during the extraction procedure, was an unexpected outcome. A previous study reported that sodium citrate diminishes the MetMb level in vacuum-packaged beef steaks when compared with that of additive-free beef steaks [24]. That study showed some differences from our study, such as their data on additive-free meat always being within the acceptable consumer criteria for MetMb during the storage period and the use of a different formula to calculate the MetMb percentage.

As also observed in our data of the control beef patties (Figure 4b), the lipid peroxidation increases during storage [17]. In our case, the M0, *D. opuntiae* extract, and BHT maintained a reduced level of TBARS with a superior performance exhibited by the *D. opuntiae* extract compared with the values exhibited in the other experimental groups. To our knowledge, there is no measurement of the lipid peroxidation level in meat products fortified with a *D. opuntiae* extract. One study reported a reduction in TBARS upon the use of carmine in pork sausage samples [5]. Again, the beef meat fortified with the M0 extract (sodium citrate) showed an unexpected outcome at day 12, a high level of TBARS, compared with the results of a previous study using vacuum-packaged beef steaks [24]. The differences between that study and our study are the very low levels of TBARS and MetMb reported in that study in their additive-free meat samples throughout the storage period. Finally, the present study evaluated important meat quality parameters for beef patties, including key predictors for consumer acceptability, such as the redness and MetMb content. It is clear that further evaluations are necessary to increase the actual evidence, such as sensory tests. Nevertheless, our study is the beginning of the use of a primary pest of cactus, such as the wild cochineal, as a preservative for the meat industry.

## 5. Conclusions

For the first time, we demonstrated the value of the insect pest *D. opuntiae* using an antioxidant extract obtained from it for the preservation of beef patties. Our study showed the carminic acid content and antioxidant properties in vitro of the extract alongside their beneficial effects against the discoloration and oxidation of proteins and lipids in beef meat during their storage. Additionally, our study included an easy and accessible procedure to obtain the extract from the insect to use directly in meat products.

## Figures and Tables

**Table 1:** Color parameters and antioxidant capacity obtained from different meat groups.

Parameter	Time (Days)	Co	M0	M3	BHT
Lightness (L*)	0	49.1 ± 3.7	43.1 ± 0.5 ^a^	47.8 ± 1.2 ^b^	43.0 ± 0.7 ^a^
	6	48.4 ± 3.1	41.8 ± 0.8 ^a^	46.7 ± 0.6 ^b^	40.7 ± 1.0 ^a^
	12	48.5 ± 4.6	41.6 ± 0.9 ^a^	46.1 ± 1.2 ^b^	40.9 ± 1.5 ^a^
Redness (a*)	0	27.5 ± 4.2	34.1 ± 1.7 ^b^	33.0 ± 1.8 ^b^	39.2 ± 0.4 ^a^
	6	18.7 ± 2.4 ^A^	17.8 ± 3.3 ^A^	18.1 ± 2.7 ^A^	22.6 ± 2.1 ^aA^
	12	15.5 ± 4.8 ^A^	15.0 ± 3.7 ^A^	15.2 ± 2.4 ^A^	17.1 ± 5.6 ^B^
Yellowness (b*)	0	18.4 ± 1.5	20.1 ± 0.7 ^c^	19.9 ± 1.5 ^b^	22.4 ± 0.4 ^a^
	6	14.9 ± 0.9 ^A^	14.0 ± 0.8 ^A^	14.5 ± 0.9 ^A^	14.3 ± 1.2 ^A^
	12	14.0 ± 0.9 ^A^	13.5 ± 0.5 ^A^	13.7 ± 0.9 ^A^	13.4 ± 0.4 ^A^
AC (µg AAE/g sample)	0	108.3 ± 6.4	111.0 ± 6.1	115.1 ± 6.5	110.9 ± 5.5
	6	113.9 ± 8.6	110.7 ± 5.2	106.3 ± 12.3	115.6 ± 11.1
	12	106.0 ± 11.0	106.6 ± 7.5	109.5 ± 8.0	118.0 ± 12.8 ^a^

Each asterisk (*) that follow a letter is part of the name of each color space parameter. Superscript lowercase letters indicate differences within the same day, while superscript uppercase letters indicate differences between days. For lightness data from the same day: ^a^ *p*-value of <0.001 versus Co group. ^b^ *p*-value of <0.01 versus M0 and BHT groups. For redness data from the same day: ^a^ *p*-value of <0.05 versus all the groups. ^b^ *p*-value of <0.01 versus Co group. For yellowness data from the same day: ^a^ *p*-value of <0.001 versus Co and M0 groups. ^b^ *p*-value of <0.05 versus Co and BHT groups. ^c^ *p*-value of <0.01 versus Co group. For redness and yellowness data on different days: ^A^ *p*-value of <0.001 versus day 0. ^B^ *p*-value of <0.05 versus day 0 and 6. For AC data from the same day: ^a^ *p*-value of <0.05 versus the Co group. AC, antioxidant capacity; Co, meat without additive; M0, meat with M0 extract added; BHT, meat with BHT added; M3, meat with M3 extract added.

## Data Availability

The datasets used and analyzed during the current study are available from the corresponding author upon reasonable request.
